# Substitution of care for chronic heart failure from the hospital to the general practice: patients’ perspectives

**DOI:** 10.1186/s12875-017-0688-z

**Published:** 2018-01-09

**Authors:** J. A. Wildeboer, A. R. T. van de Ven, D. de Boer

**Affiliations:** 1 VU University Amsterdam, De Boelelaan 1105, 1081 HV Amsterdam, Netherlands; 2 St. Anna Hospital, Bogardeind 2, 5664 EH Geldrop, Netherlands; 3 NIVEL, Otterstraat 118-124, 3518 CR Utrecht, Netherlands

**Keywords:** Substitution, Patients’ attitudes, Chronic heart failure

## Abstract

**Background:**

Shifting care from the secondary to the primary system may present an opportunity to ensure that the increasing number of patients with chronic heart failure (CHF) receive high-quality care while containing costs. However, shifting from secondary to primary care might seem radical to patients. A qualitative insight into patients’ issues, preferences, expectations and needs may help arrange a smooth transition from secondary to primary care for CHF patients. The aim of this exploratory study is therefore to gain insights into the way CHF patients in secondary care perceive the possibility of substitution of CHF care from secondary to primary care.

**Methods:**

In total, fifteen semi-structured interviews were conducted with CHF patients. Topics discussed during the interviews were the advantages and disadvantages, attitudes of patients, preferences regarding the substitution and trust in the GP and cardiologist. A thematic analysis was performed.

**Results:**

The minority of the patients welcomed the idea of substitution. Against that, the majority of the patients had various concerns. This attitude was mainly influenced by two main themes, confidence and security and accessibility. Most patients had more confidence in secondary than in primary care because of the greater level of knowledge and more possibilities for examination in secondary care and because of good relationships and positive previous experiences in secondary care. Patients also indicated that the general practice is geographically more easily accessible than the hospital.

**Conclusion:**

Patients had various concerns regarding the substitution of care for chronic heart failure. Addressing these concerns by informing them appropriately may contribute to a smooth and patient-friendly substitution from secondary to primary care. The fears and needs of patients could also be taken into account by policymakers when optimising the way substitution is organised, or when substitution is introduced.

**Electronic supplementary material:**

The online version of this article (10.1186/s12875-017-0688-z) contains supplementary material, which is available to authorized users.

## Background

Heart failure is a chronic condition with a high prevalence and a high morbidity and mortality [1, 2]. Worldwide, the prevalence of chronic heart failure (CHF) is 23 million [3]. The prevalence of CHF has increased over recent decades and is expected to increase further in the coming years [1, 4, 5]. The costs of CHF are considerable [6, 7]. In western society, CHF comprises 1–2% of all healthcare costs. The majority of these costs are for frequent, long-lasting and repeated hospitalisations [7].

Changes in healthcare and treatment for CHF are needed in order to ensure that the increasing number of patients with CHF receive high-quality care and to reduce the associated costs [8, 9]. Substitution, which optimises the care process by shifting care is a change that could be implemented to handle this problem [8, 9]. Substitution is defined as “the replacement of all or part of an existing healthcare facility by all or part of another facility, in which the original function will be fulfilled for a similar patient population” (p.9) [9].

Currently, CHF patients are under treatment in secondary care. Stable CHF patients visit their heart failure nurse and cardiologist both twice a year for a routine check. The Dutch government is interested in strategies to decrease the healthcare costs and to provide high quality of care [10], and substitution is one of these strategies. Substituting CHF care to primary care may be a more sustainable and a cost-efficient approach of treating stable CHF patients. Treating patients in primary care is less expensive than treating patients in secondary care due to the specialist and more expensive care that is provided in secondary care [9]. However, a solid primary care is necessary in order to treat patients in primary care. In the Netherlands, the primary care is well developed and organised and substitution of CHF care for stable patients would be possible.

The substitution approach is still evolving in the Netherlands and has recently been applied on a small scale in a number of regions and hospitals in the treatment of CHF [11, 12]. In the case of a shift from secondary to primary care, the general practitioner (GP) becomes the central care provider instead of the medical specialist. This might seem to be a radical shift to patients who are currently being treated in secondary care. To date only quantitative studies have been done to investigate patients’ preferences for substitution. Those studies showed that patients prefer medical specialists for complex treatments and GPs for less complex treatments [9, 13]. A qualitative insight into patients’ issues, preferences, expectations and needs relating to substitution of care will increase our understanding of how patients perceive this. In addition, such understanding can help to optimise the way in which substitution is organised from the patients’ perspective, which can help improve the quality of care. The aim of this exploratory study is therefore to gain insights into the way CHF patients in secondary care perceive the possibility of substitution of CHF care from secondary to primary care.

### Research question

How do chronic heart failure patients in secondary care perceive the possibility of substitution of their CHF care from secondary to primary care?

## Method

### Study design

Semi-structured interviews were conducted in this qualitative study to investigate how patients perceive substitution of CHF care. A topic list that was covered in the interviews included the advantages and disadvantages of substitution, attitudes of patients towards substitution, the relationships with the cardiologist and GP, trust in the cardiologist and GP, and patients’ preferences in the matter (Additional file 1). Furthermore, probing questions were prepared. The topic list for the interviews was based on a theoretical model by Sixma et al. (1998) and six elements that determine quality of care according to the WHO [14, 15]. This conceptual model states that patients’ perspectives can be identified by assessing the needs and expectations of patients. By identifying the needs and expectations of the six elements that define quality of care (effectivity, efficiency, accessibility, acceptability equity and safety), patients’ perspectives towards the substitution from secondary to primary care were identified.

### Participants and data collection

A group of patients were recruited from the population of CHF patients at the St. Anna hospital in Geldrop in the Netherlands. Patients received an invitation letter followed by a phone call within a week. Convenience sampling was used to select participants. A total of 18 patients were approached and 15 patients agreed to participate. Participants were recruited until data saturation was achieved [16]. This group of participants was selected from a list of patients who had an appointment with the heart failure nurse at several days in 2016. Nine interviews were scheduled following a doctor’s appointment in the hospital and six interviews were conducted by phone due to logistic reasons and patients’ preferences. An independent researcher, who did not have a relation with the participants, conducted the interviews and to ensure confidentially only the researcher and the participant were present in the room. At the beginning of the interview, the idea of transferring CHF care to primary care was explained to patients. Essentially, this transfer of care would mean that patients visit the GP for their CHF care and only see the specialist in the hospital through a referral from the GP. All participants gave approval for the interview to be recorded and signed an informed consent form. Additionally, patients filled in a seven-question questionnaire about demographic information. After the interview, a summary of the interview was sent to all participants for verification. The majority of the participants approved the summary and in a single case several adjustments were made. All interviews lasted around 30 min.

### Data analysis

The data analysis for this study was based on thematic analysis [17]. The recordings of the interviews were transcribed verbatim, using Express Scribe Transcription Software. These transcripts were coded by using MAXQDA 12. The conceptual model was used as a starting point of the analysis of the transcripts. The transcripts were coded by using the concepts of the conceptual framework. Thereafter, open coding was used to identify the important aspects within each concept [17, 18]. The first three interviews were coded separately by the main researcher and a colleague. Discrepancies between the researchers were discussed, which resulted in a number of small changes in the coding system. The themes, deriving from the open coding, were named and relationships between the themes were identified in consultation with the main researcher and a supervising researcher [18].

## Results

### Research population

The participants were 15 CHF patients from St. Anna Hospital. Nine patients (60%) were male and six patients (40%) were female. The average age was 74, ranging from 65 to 81. All patients were born in the Netherlands. Patients described their state of health on average as moderate. Four patients (27%) did not have any other conditions in addition to CHF and eleven patients (73%) had one or more comorbidities such as diabetes, other heart and vascular diseases, renal insufficiency and pulmonary hypertension. These characteristics of the research population are representative of the entire population of CHF patients in the Netherlands [19–21]. The majority of the patients were classified according the New York Heart Association (NYHA) classification in class II and a minority in class III. Class II refers to slight limitation of physical activity and comfortable at rest. Class III refers to marked limitation of physical activity and comfortable at rest [22]. No different results were found in the face-to-face or telephonic interviews.

### General attitude towards repositioning

The majority of the patients had a negative attitude towards substitution of care. These patients had a better relationship with their cardiologist and/or specialist heart failure nurse than with their GP, which let them feel more at ease in the hospital. Furthermore, these patients had more confidence in the cardiologist because of the specialisation in heart conditions, and patients felt that their heart condition was a severe condition for which they preferred a specialised physician.

In contrast, a number of patients was positive about the substitution from secondary to primary care for the treatment of CHF. They saw the substitution as a natural form of progress and reasoned that the hospital is still always there if necessary. These patients also saw the geographical proximity of the GP as an advantage. Furthermore, these patients indicated that a general practice had a friendlier ambience than a hospital, which made them to feel more at ease. Despite the fact that some patients were positive, they also had some requirements for substitution of care. First, a yearly check-up with the cardiologist in addition to the check-ups in primary care. Furthermore, patients thought they should get the same amount of time in primary care as in secondary care for a consultation. Currently, a half-hour check-up consultation in secondary care was perceived as pleasant by patients. Usually patients have a ten-minute consultation in primary care, which is felt to be too short for a check-up consultation about heart failure. Lastly, the possibility of examination at the general practice was mentioned. It appeared that the possibility of having an ECG or ultrasound in primary care would provide a sense of security for patients:

*“I’d prefer an ECG once a year, just for a bit of extra safety and confidence. My health status isn’t that good, so an ECG would be reassuring to have once a year. Yes, that would be a point of concern for me if I have to go to the GP instead of the hospital.”* (Interview 1)

Two themes that influenced the attitude towards the substitution of heart failure care arose from the data. Security and confidence turned out to be the main theme, appearing to consist of several subthemes. The second theme that arose was accessibility.

### Confidence and security

Confidence and security appeared to be the most important concepts for patients in influencing the way they looked at substitution of heart failure care. Most patients had more confidence in their cardiologist than their GP in the specific case of heart failure. In addition, patients are currently treated for CHF in secondary care, which made them familiar with that situation. Accordingly, patients do not know what to expect from the new situation, whereas they have faith in the current situation. These broad observations became manifest in various ways in the following subthemes.

### Knowledge

The knowledge and expertise of a cardiologist made patients feel safe. Patients had the idea that a lack of knowledge could mean that a GP or practice nurse cannot help them as effectively and may still refer them to the cardiologist. Visiting the GP or practice nurse as well as the cardiologist would be an additional burden on patients:

*“I prefer the hospital because in the hospital I trust the people they are more specialised in heart conditions than a GP. They [GP] haven’t studied for a specific part of the body, and that doesn’t give me confidence… For cases like this, I have more confidence in a specialised physician than in a GP.”* (Interview 14)

### Burden of disease

Most patients see heart failure as a condition that is too severe for a GP or practice nurse. Patients therefore preferred a specialised caregiver who treats CHF. This was particularly the case for patients in unstable situations, although it was not limited to unstable patients. Patients in stable situations also indicated this as a point of concern:

*“I have a serious condition and I think that it is too severe to see a GP or practice nurse for. I think just going to the cardiologist is better because heart failure is a serious illness.”* (Interview 2)

### Relationship with care provider

The relationship with the care provider had a major influence on the extent to which patients trust them. Because the good, long-term relationships that most patients had with their cardiologists, these patients trusted their cardiologists more than their GPs and practice nurses. Patients thought that the relationship with their cardiologist cannot be built up with someone else. One of the patients described it as follows:

*“The cardiologist and the specialist heart failure nurse are on a pedestal for me and no one should touch that. I’d never want to lose those two. I trust them completely. They mean so much to me, I believe them instantly.”* (Interview 7)

On the other hand, a number of patients had a better relationship with their GP than with their cardiologist. The ambience of a general practice was friendlier and more accessible than a hospital. Patients had been seeing their GP for many years and also knew their GP from the community where they lived. Patients who perceived a better relationship with their GP than with their cardiologist were generally more open to the idea of repositioning their heart failure care.

### Past experiences with care provider

Past experiences with a care provider also influenced the degree of confidence patients had in a care provider. Except for one patient, all patients had very positive experiences with the heart failure clinic. The nurse at the heart failure clinic had enough time to answer questions and if necessary the opportunity to consult a cardiologist directly, who was available at the hospital. Furthermore, the majority of these patients indicated that they were approached with a touch of humour, which patients perceive as pleasant. The one patient who had not had good experiences with the heart failure clinic was dissatisfied with the accessibility of the heart failure clinic by phone.

A few patients had less pleasant experiences with their GP regarding their heart condition. These patients reported that they were not redirected appropriately to the hospital or were misdiagnosed. These past experiences reduced patients’ confidence in their GPs with respect to their heart conditions:

*“Yes, yes, I really didn’t have much confidence in him [GP] because of things I’ve experienced before and that incident it was only confirmed it more. They don’t inspire confidence.”* (Interview 15)

In contrast, one patient mentioned that his GP responded well in an acute situation when an acquaintance had heart problems. This increased the confidence of this patient in his GP with respect to heart conditions.

### Communication between care providers

Another aspect that affected trust is the communication between care providers. Patients felt that access within the hospital to various different care providers was easier, which gave them a sense of security. Patients felt safer if the cardiologist could get involved very quickly. This was experienced more in the hospital than at the general practice. However, some patients indicated that the connection between the GP and the hospital was also quick:

*“I think the GP is capable enough of calling an ambulance if something goes wrong, and then you will be in the hospital very quickly… Those people [care providers] have contact very quickly and they consult each other directly if something’s wrong.”* (Interview 6)

In addition, patients said they should have the opportunity to be redirected from the GP back to the hospital, even if they were at the GP for treatment of CHF. This generated feelings of confidence. However, some patients were a bit concerned that referral to the hospital would mean appointments with several different physicians, which would be too much for them.

### Possibilities for examination

The extra opportunities for examination in secondary care created feelings of confidence in the patients. Patients felt there were more possibilities for examinations in a hospital, where it is possible to get an ECG, ultrasound, MRI and other medical examinations.

*“I think the cardiologist ultimately has more possibilities for examinations than a GP, I think. If the cardiologist thinks something’s wrong, he can immediately decide to do an ECG or ultrasound, and at the GP that’s more difficult.”* (Interview 13)

Patients did not need examinations every time they visit the hospital, but they particularly preferred having the possibility as an option. This was seen as being available in a hospital but not in a general practice.

#### Accessibility

A frequently mentioned concept when talking about substitution of care is accessibility, in particular geographical accessibility. All patients who did not live in the same town as the hospital said that the general practice is geographically more easily accessible. A general practice was often in the same village where patients lived. This made it possible for patients to get to the GP on foot or by bike, if they were physically able:

*“Ohhh yes, the trip to the GP instead of the journey to the hospital, that’s an advantage, without a doubt. For the GP, I can just go by bike but for the hospital I have to go by car… For me the bike is just much easier.”* (Interview 5)

Patients who lived in the same town as where the hospital is located indicated that distance and accessibility to the hospital were not an issue for them. However, this was not limited to patients who lived in the same town.

## Discussion

### Main findings

The aim of this study was to reveal the attitudes of CHF patients towards substitution of CHF care from secondary to primary care. The majority had various concerns about shifting CHF care, while a minority appeared to be more open to the idea of shifting their heart failure care to primary care. The considerations patients reported regarding the way they saw substitution could be attributed to confidence and security and accessibility. The confidence and security domain appeared to consist of several sub domains, including the level of knowledge of the care provider, the burden of disease, the relationship with the care provider, past experiences with care providers, communication between care providers and possibilities for examination. The main concepts, confidence and security and accessibility are in accordance with the conceptual model that was used in this study. However, the other elements were hardly raised by patients considering substitution of CHF care. Furthermore, the needs and perspectives of patients according to the model of Sixma et al.*,* (1998) towards these two concepts were identified in this study.

The fact that confidence and security appeared to play a large role in patients’ considerations regarding substitution of their care for CHF is consistent with previous literature. Firstly, confidence and security are important issues for patients in general [23, 24]. Secondly, for repositioning in particular, a study by Bodegom-Vos et al. (2013) demonstrated that patients’ preferences for substitution of care are significantly affected by the type of medical intervention. Patients prefer medical specialists for more complex procedures, which would be the case for CHF. Lastly, the perceived severity of the condition – which would be high for CHF patients – may also partly explain why confidence and security are considered important in the context of repositioning [9, 13].

Within the main concept of security and confidence, several subdomains were identified in the present study that are consistent with previous research. For instance, a study by Tarrant et al. (2003) showed that when the relationship between physicians and patients is better, patients also feel more confident and safer with the physician [25]. This is in line with patients’ experiences in this study. In addition, positive experiences in the past with a care provider helped patients trust a care provider [26]. As such, a good relationship with a GP and pleasant experiences were associated with patients being more open towards repositioning. Furthermore, patients appeared to associate the possibilities for examination with confidence, as they reported feeling more at ease if they knew that additional examinations could be performed if necessary. This is also demonstrated in the literature [27, 28]. Additionally, the association that patients reported between communication among care providers and trust is confirmed by the literature [29]. Patients trusted care providers more when there was clear communication between care providers.

### Strengths and limitations

A strength of the study is that respondent validation was obtained in this study. This provided the opportunity to validate the researcher’s interpretation of the data. This improved the validity of this study. Furthermore, this study is the first qualitative study, as far as we are aware, that extensively investigates patients’ attitudes towards substitution of CHF care. To date, only quantitative studies have been conducted to investigate this [9, 13].

This study has also a limitation, all patients were treated by the same specialised heart failure nurse, which is a form of selection bias [30]. This could have influenced the results, as it was found that past experiences influenced how patients felt about repositioning. Additionally, both a strength and a limitation of the present study is that the severity of the CHF patients differed. The majority of the patients was classified according to the NYHA classification in class II, which are relative stable CHF patients, and a number of patients was classified in class III, which are more unstable patients. However, the substitution of CHF care that is being developed is currently focussed on patients in class II. This means that we collected the perspectives of a broader patient population than those for which the substitution is currently being developed. This is an advantage for the richness and diversity of the perspectives we collected, but is also a limitation as some concerns raised by patients in our study might be slightly pronounced due to the severity of their condition.

### Potential implications

This study is to our knowledge the first qualitative study that investigated patients’ attitudes to substitution for chronic conditions. Policymakers and medical professionals can take the fears and needs of patients into account when optimising the way in which substitution is organised, or when substitution is introduced. These policies should include patients’ needs for confidence and safety, accessibility, time constraints in primary care and the relationship between patients and clinicians. Furthermore, security and confidence appeared to be a major influence on how patients’ see substitution. Trust in primary care should therefore be taken into account when optimising the way in which substitution is organised. The aim of substitution is to ensure that an increasing number of patients receive high-quality of care and to reduce the associated costs. This study identified the challenges from a patients’ perspective of substitution. In the upcoming years, these challenges should be taken into account and evaluated, since CHF patients have often fears and uncertainties about the development of their condition. Furthermore, these challenges should also be identified for other chronic conditions, which could possibly be suitable for substitution. This is important to consider in future policies on primary and secondary care to make the healthcare system as sustainable as possible. This study is a beginning towards a sustainable health system for CHF patients.

From literature, it is known that giving patients self-control will create feelings of security and confidence towards care givers [31, 32]. It is therefore likely that giving patients self-control in the way substitution is organised means that patients will have more trust in primary care and thereby be more positive towards repositioning. One option for giving control to patients is offering them a chance to visit the cardiologist once a year without a referral, if they think it is necessary. A pilot should show whether this works efficiently and whether it does indeed reduce patients’ concerns about substitution.

After a discussion with various medical professionals in the field of CHF, it was concluded that most fears of patients are caused by a lack of knowledge. For instance, patients were afraid that there was a lack of knowledge and expertise in primary care. However, all GPs and practice nurses have to participate in an educational programme about the treatment of CHF before repositioning of CHF is commenced. Furthermore, patients were afraid that they would not have enough time at the GP for their check-up. However, patients also have 30 min for a consultation at their practice nurse for their CHF check-up. If patients knew this, the fear of a lack of knowledge might disappear. Patients’ fears about substitution could therefore be (at least partially) resolved by providing information to patients. The information might help patients trust primary care more and thereby give them a more positive attitude towards substitution.

## Conclusion

The present study showed that patients had various concerns about substitution of care for chronic heart failure. This means that it is important to combine changes in the organisation of care for chronic heart failure with strategies for reducing patients’ concerns. Such strategies should be based on a proper assessment of the concerns that patients have and should ideally be jointly developed by patients or patients’ representatives and healthcare professionals.

## Additional file

Additional file 1: Topic list that was used during the semi-structured interviews. (DOCX 14 kb)

## Abbreviations

CHF: Chronic Heart Failure
ECG: Electrocardiogram
GP: General Practitioner
NYHA: New York Heart Association
WHO: World Health Organisation
