# Supplementary material for: Substitution of care for chronic heart failure from the hospital to the general practice: patients’ perspectives
Source: BMC Fam Pract. 2018 Jan 9;19:8. doi: 10.1186/s12875-017-0688-z (PMC5759197; doi:10.1186/s12875-017-0688-z)
Supplement: Additional file 1: — Topic list that was used during the semi-structured interviews. (DOCX 14 kb) [file 12875_2017_688_MOESM1_ESM.docx]

**Topic list semi-structured interviews**

*General*

- How long ago have you been diagnosed with heart failure?
- How did this happen?
- How did you experience the care that was provided during that time?
  - What did you like/didn’t like?

*Explanation of substitution CHF care from secondary to primary care*

- What do you think when I tell you this?
  - Do you think it’s a good idea or not?
  - Why?
- You already mentioned an advantage/disadvantage, could you mention another advantage/disadvantage?
  - Why is this a advantage/disadvantage for you?
  - What is the most important advantage/disadvantage for you?
- What would be another advantage/disadvantage for you?
  - No, why don’t see any other advantage/disadvantage?
- What do you feel when you think about this idea?
  - What causes that you get that feeling?
- Does this idea of substitution fit your idea of good quality of care?
  - No, what should be changed to make sure that fits your idea of good quality of care?
  - Yes, what is causing that it fits your idea of good quality of care?
- If you could change anything about the idea of substituting the care to primary care, what would you like to change?
  - Could you explain why you don’t like this aspect?
  - And which aspect would you like to be the same? Why?
- What do you expect from the GP during the consultations for your CHF?
  - Could you mention an example?
  - Do you have previous experiences with this?
- What would give you confidence regarding your chronic heart failure care?
  - Why does that give you confidence?
- Do you have enough confidence in the GP or practice nurse to visit them for your CHF?
  - Why do/don’t you have this confidence?
  - Do you have previous experiences with this?
- Is it easier or more difficult for you to go the GP instead of to the hospital?
  - Do you experience this as a hurdle? Why?
- If you could mention anything that you would really like to change in the care for CHF, what would you like to change?

- Do you have any other remarks or questions?

Probing questions:

- How did you experience that?
- What would be an example of that?
- Could you explain why …?
- Why is that important to you?
- You mention that … is important to you, why?

Topics (derived from the conceptual model):

- Communication
- Accessibility
- Safety
- Trust
- Difference between hospital and GP
